# The role of pre-existing comorbidity on the rate of recovery following injury: A longitudinal cohort study

**DOI:** 10.1371/journal.pone.0193019

**Published:** 2018-02-21

**Authors:** Gabrielle Davie, Ari Samaranayaka, Sarah Derrett

**Affiliations:** 1 Injury Prevention Research Unit, Department of Preventive and Social Medicine, Dunedin School of Medicine, University of Otago, Dunedin, New Zealand; 2 Department of Preventive and Social Medicine, Dunedin School of Medicine, University of Otago, Dunedin, New Zealand; Hunter Holmes McGuire VA Medical Center, UNITED STATES

## Abstract

Understanding the role of comorbidity in recovery following injury is an important challenge given the increasing prevalence of multimorbidity (2 or more comorbidities) in many countries. The Prospective Outcomes of Injury Study recruited 2856 injured 18–64 year olds that had registered for entitlements with New Zealand’s universal no-fault injury insurer. Recovery, or lack of, in this longitudinal cohort was measured using the World Health Organization Disability Assessment Schedule at 3, 12 and 24 months post-injury. Twenty-one pre-existing chronic conditions were used to identify comorbidity. To investigate whether rates of recovery differed by pre-injury comorbidity, the interaction between time and comorbidity was modelled using Generalised Estimating Equations. Of 1,862 participants with complete data, the distribution reporting none, one comorbidity, or multimorbidity pre-injury was 51%, 27%, and 21% respectively. Longitudinal analysis estimated no difference (log odds per year 0.05, 95% Confidence Interval -0.17 to 0.27) between the rate of change of disability for those with one pre-injury comorbidity compared to those with none. Those with pre-injury multimorbidity had significantly slower reduction in disability over time than those with no pre-injury comorbidity (log odds per year 0.27, 95% Confidence Interval 0.05 to 0.48). In a working age cohort, the rate of recovery in the 24 months following injury was similar for those with none or one pre-existing comorbidity and significantly slower for those with multimorbidity. It is important that further research explores mechanisms driving this, and that researchers and health providers identify and implement better supports for injured people with multimorbidity.

## Introduction

Internationally injury is a leading cause of hospitalisation and global estimates indicate it accounts for 10% of the “burden of disease” [1–3]. Given that injury, and subsequent disability, inflict a high burden on individuals and society, recovery from injury is under researched and more comprehensive population-level outcomes based research has been called for [4].

In many countries the increasing prevalence of multimorbidity (two or more comorbidities) is adding weight to the importance of understanding the role of comorbidity in recovery following injury [5]. Pre-existing comorbidity has been reported by cohort studies as an important predictor of poor outcomes at different time points following injury [6–9]. One of the few longitudinal studies that has explored the relationship between comorbidity and the rate of recovery following injury reported hospitalised Dutch patients with none or one pre-injury comorbid conditions reached their maximum health status after 9 months whereas those with multimorbidity made considerable improvement in functional outcomes between the 9 and 24 month assessments [10]. In a study of adult major trauma survivors the rate of return to work/study in the two-years following injury was found to be lower for those with serious pre-existing comorbidities compared to those with no comorbidities [11].

Data collected in the Prospective Outcomes of Injury Study (POIS) offers the opportunity to add to the scant research on pre-existing comorbidity and the rate of recovery following injury by using the World Health Organisation Disability Assessment Schedule (WHODAS) to assess recovery [12]. In addition, unlike other injury outcome studies, participants from POIS were recruited from a general injury population (rather than from an Emergency Department or hospital) so the cohort includes a diverse range of injury diagnoses and causes [13].

The objective of this research was to compare rates of recovery (according to a measure of disability) over 24 months following injury for those with no pre-existing comorbid conditions, one comorbidity and multimorbidity from within a general injury population. This study will add important knowledge to the evidence base helping researchers and health providers identify and implement better supports for those with poorer recovery rates.

## Methods

### Study sample and recruitment

This study uses existing data from POIS, a prospective longitudinal cohort study of 2856 people injured between June 2007 and May 2009. The study was granted ethical approval by New Zealand’s Multi-Region Ethics Committee (MEC/07/07/093). Recruitment was via the entitlement claims register of New Zealand’s no-fault injury compensation insurer, the Accident Compensation Corporation (ACC). Entitlement claimants have injuries serious enough to potentially require compensation and/or support for returning to independence (e.g. income support, home support or assistance with returning to work) in addition to receiving cover for their medical treatment. [14] Claimants whose injuries resulted from self-harm or sexual assault were excluded. Participants, aged 18–64 years and residing in one of five regions of New Zealand, were interviewed up to four times since injury. In the phone interviews participants’ identities were confirmed by comparing responses with available information such as date of birth and details of injury. After receiving comprehensive information about the study, all participants granted oral consent documented by interviewers and were sent paper copies of the consent form for their records. Participants received a $NZ10 Motor Trade Association (MTA) voucher for each completed interview. Other details of the recruitment process have been described elsewhere [13]. This paper uses data collected from interviews held at three months (median = 3.2; interquartile range, IQR = 2.5, 4.2), 12 months (median = 12.3; interquartile range, IQR = 12.0, 12.9) and 24 months (median = 24.4; IQR = 24.1, 25.1) after injury.

### Outcome measure

Recovery was measured using the brief World Health Organization Disability Assessment Schedule (WHODAS II 12-item) that assesses activity limitations and participation restrictions over the past 30 days along six dimensions (understanding and communication, self-care, mobility, interpersonal relationships, work/ household roles, and community roles) [15]. Responses to the 12 items were combined using the simple summed method. If a single response was missing the rounded mean of the remaining 11 items was imputed; if participants were missing more than one item, no score was calculated [9]. WHODAS was administered at the 3, 12 and 24 month interviews. At the first interview participants were also asked to report their WHODAS status for the 30 days prior to their injury. WHODAS scores which have a possible range from 0–48 were categorised with participants having ‘disability’ if their WHODAS score was ≥10, and as having ‘no (or lesser) disability’ if their score was <10 [9, 16, 17].

### Explanatory variable

At the first interview, participants were asked to identify, from a list of 21 conditions, any that had been diagnosed by a doctor before their injury and which had lasted or were expected to last for more than six months. These conditions, listed in Table 1, have been used by New Zealand’s Ministry of Health [18]. The number of pre-injury conditions reported by participants was categorised into none, one comorbidity or two or more (multimorbidity).

**Table 1:** Prevalence and type of pre-existing comorbid conditions in an injured population (N = 1,862).

Pre-existing comorbid conditions	No comorbidities		One comorbidity		Multi- morbidity	
	n	%	n	%	n	%
Overall (row %)	957	51.4	510	27.4	395	21.2
Comorbid conditions (column %):						
Neck/back disorder			117	22.9	191	48.4
Asthma			101	19.8	119	30.1
Migraine			61	12.0	122	30.9
Arthritis			50	9.8	120	30.4
Diabetes			39	7.6	49	12.4
Depression			31	6.1	131	33.2
Heart disease			29	5.7	59	14.9
Irritable bowel			25	4.9	56	14.2
Cancer			13	2.5	22	5.6
Anxiety			12	2.4	100	25.3
Stomach ulcers			9	1.8	33	8.4
Osteoporosis			7	1.4	37	9.4
Epilepsy			6	1.2	15	3.8
Stroke			4	0.8	18	4.6
Bipolar disorder			3	0.6	10	2.5
ME/CFS*			0	0	16	4.1
COPD**			0	0	7	1.8
Other specific comorbid conditions:						
Bronchitis/Emphysema, Multiple sclerosis, Motor neurone disease or Schizophrenia			3	0.6	26	6.6

*Myalgic Encephalopathy/Chronic Fatigue Syndrome

**Chronic Obstructive Pulmonary Disease

### Covariates

Previous POIS analyses were used to inform the selection of covariates from questions asked at the first interview [7, 9, 17]. Participants reported age, gender and ethnicity based on New Zealand Census questions [19]. Prioritisation of ethnicity for those who provided multiple ethnicities was undertaken resulting in each participant being included in only one of the following groups: Māori (New Zealand’s indigenous population), Pacific, sole New Zealand European, and Other [20]. In addition to categories of Body Mass Index (BMI)<30 and BMI≥30, an “undisclosed BMI” category was used so that participants who did not report height and/or weight could be included [17, 21]. Household income was coded as ‘adequate’ if participants reported ‘just enough’, ‘enough’ or ‘more than enough’ total pre-injury household income to meet their everyday needs; or ‘inadequate’ if they reported ‘not enough’ income [22]. Post-injury access to healthcare services was obtained by asking if they had trouble getting to or contacting health services; ‘yes’ and ‘mixed’ responses were compared to those who reported ‘no’ trouble. Participants that responded ‘yes’ or ‘maybe/possibly’ to the question “At the time, did you feel the injury was a threat of severe longer-term disability to you?” were compared to those responding ‘no’ [8]. Participants admitted to hospital or treated at an Emergency Department for at least 3 hours within 7 days of the injury event (‘hospitalised’) were identified using probabilistic linkage to the National Minimum Dataset [23]. New Injury Severity Scores (NISS) were derived for each participant [24]. NISS was categorised as 1–3 (least severe), 4–6 and >6 (most severe) for descriptive tables; model-based analysis included NISS as a continuous variable.

### Analysis

Bivariate analyses were undertaken; χ^2^ p-values and 95% Confidences Intervals (CIs) have been presented alongside frequencies and percentages. A repeated measures dataset with three rows per person (one per interview) was analysed using Generalised Estimating Equation (GEE) population-averaged models specifying a binomial distribution with a logit link and an unstructured correlation matrix [25]. Robust standard errors adjusted for clustering by participant. Time (the number of days between date of injury and date of interview) was included as a continuous variable with a quadratic term for time ((Time)^2^) used to address non-linearity. Rather than using change in disability from pre-injury as the outcome measure, differences in pre-existing disability were accounted for by including pre-injury WHODAS as a covariate when estimating post-injury recovery. Inclusion of the interaction between time and comorbidity group provide an estimate from which to assess whether rates of recovery differed by prior comorbidity. To help interpret the regression coefficients, model estimates were used to predict disability for each individual and from these, predicted average proportions with disability in the pre-injury comorbidity groups were obtained.

For comparisons between physical and mental comorbidities the following were considered mental health conditions: bipolar disorder, schizophrenia, depression and anxiety.

Analysis was undertaken using Stata 13 [26].

## Results

The injuries sustained by the 2,856 POIS participants were varied with 39% receiving multiple types of injury; of those with a single injury type, sprains and strains, and fractures were most prevalent [13]. WHODAS scores at each of the 3, 12, and 24 month interviews and pre-injury WHODAS could be derived for 1,903 (67%) participants. Of these, 41 were excluded as no response on pre-injury comorbidity was provided, leaving 1,862 participants A comparison of this subset with the remainder of the POIS cohort indicates those not included were more likely to: be older, male, not willing to disclose their BMI, of Māori or Pacific ethnicity, to have inadequate income, and to report their injury to be of potential threat of disability (S1 Table). There was no evidence to suggest that the distribution of number of pre-existing comorbidities differed between those included and those not included.

Of the remaining 1,862 participants, 51% reported no pre-injury comorbidity, 27% reported one and 21% reported two or more (Table 1). Of the 395 with multimorbidity, 51% had two pre-injury comorbidities, 28% had three, 11% had four and 7% had five with the remaining 13 people having between six and nine pre-injury comorbidities.

Of those reporting one pre-injury comorbidity, asthma (20%) and disorders of the neck and back (23%) were the most common (Table 1). Of those with pre-injury multimorbidity almost half had disorders of the neck/back.

Of those with no pre-injury comorbidities, 63% were male whereas the ratio of males to females among those with multimorbidity was more even (Table 2). Almost a third (30%) of those with multimorbidity were 55–64 years old at the time of injury compared to 15% of those with no prior comorbidities. Those who self-identified as sole New Zealand European accounted for proportionately less of those with no prior comorbidities compared to those with multimorbidity. The percentage with a BMI ≥30 increased as the number of comorbidities increased. Similarly low percentages of pre-injury disability were observed for those with none or one comorbidity at the time of injury (2% & 3% respectively) whereas of those with multimorbidity 15% reported pre-injury disability.

**Table 2:** Number and distribution of pre-existing comorbid conditions in an injured population (N = 1,862) by demographic, pre-injury and injury-related characteristics.

Characteristics	No comorbid conditions		One comorbidity		Multi- morbidity		p-value
	(N = 957)		(N = 510)		(N = 395)		
	n	%	n	%	n	%	
Sex							
Male	600	62.7	288	56.5	193	48.9	<0.001
Female	357	37.3	222	43.5	202	51.1	
Age at injury (years)							
18–24	126	13.2	52	10.2	22	5.6	
25–34	223	23.3	76	14.9	55	13.9	
35–44	219	22.9	103	20.2	95	24.1	
45–54	244	25.5	146	28.6	105	26.6	
55–64	145	15.2	133	26.1	118	29.9	<0.001
Ethnicity*							
Maori	153	16.0	85	16.7	72	18.2	
Pacific	59	6.2	19	3.7	10	2.5	
Sole NZ European	544	56.8	319	62.5	264	66.8	
Other	200	20.9	87	17.1	49	12.4	<0.001
Body Mass Index							
<30	730	76.3	365	71.6	255	64.6	
≥30	200	20.9	129	25.3	125	31.6	
unstated	27	2.8	16	3.1	15	3.8	0.001
Income adequacy							
adequate	870	90.9	468	91.8	350	88.6	
inadequate	81	8.5	39	7.6	42	10.6	0.3
Prior disability							
no	937	97.9	493	96.7	335	84.8	
yes	20	2.1	13	2.5	60	15.2	<0.001
New Injury Severity Score							
1–3	355	37.1	212	41.6	184	46.6	
4–6	479	50.1	222	43.5	159	40.3	
>6	99	10.3	56	11.0	40	10.1	0.009
Hospitalised within 7 days for injury							
yes	250	26.1	131	25.7	90	22.8	
no	707	73.9	379	74.3	305	77.2	0.4
Trouble accessing healthcare services							
yes or mixed	87	9.1	52	10.2	37	9.4	
no	861	90.0	455	89.2	354	89.6	0.8
Perceived threat of disability							
yes or maybe/possibly	355	37.1	185	36.3	173	43.8	
no	587	61.3	316	62.0	216	54.7	0.04

*Prioritisation of ethnicity for those participants who provided multiple ethnicities followed a Statistics New Zealand algorithm. [20]

A higher percentage of lower severity injuries (NISS 1–3) was observed in those with multimorbidity compared to those with no pre-injury comorbidity (48% and 38% respectively). Differences between the comorbidity groups and income adequacy, trouble accessing healthcare services and hospitalisation within seven days of the injury were less apparent. Of those with none or one pre-existing comorbidity, 37–38% perceived their injury to be of severe long-term disability compared to 45% of those with multimorbidity.

Of those with no pre-existing comorbidity, 37% (95%CI 34% to 40%) were experiencing disability 3 months following injury (Table 3). This dropped to 11% (95%CI 9% to 13%) by 12 months post-injury with a slight reduction to 9% experiencing disability (95%CI 7% to 11%) in the following 12 months. The magnitude and pattern of disability for those with one pre-existing comorbidity was similar. Of those with multimorbidity, more than half were experiencing disability 3 months following injury; by 12 months this had reduced to 27% (95%CI 23% to 32%); still significantly higher than among those with no or one pre-injury comorbidity. At 24 months post-injury a quarter of the multimorbidity group were still experiencing disability, again higher than for the other comorbidity groups.

**Table 3:** Percentage with disability (WHODAS≥10) at 3, 12 and 24 months post-injury by pre-existing comorbidity (N = 1,862).

Pre-existing comorbidity	Months post-injury	% disabled	95% CI
No comorbidities (n = 957)	3	37.2	(34.1, 40.4)
	12	10.6	(8.7, 12.7)
	24	8.9	(7.2, 7.2)
One comorbidity (n = 510)	3	39.8	(35.5, 44.2)
	12	11.4	(8.7, 14.5)
	24	10.8	(8.2, 13.8)
Multimorbidity (n = 395)	3	51.9	(46.8, 56.9)
	12	27.1	(27.1, 31.8)
	24	24.6	(24.6, 29.1)

Estimates were obtained from two separate GEE models estimating the logit of disability over time; both included an interaction term between time and comorbidity group and a quadratic term for time (Table 4). Model 1 adjusted for pre-injury WHODAS whereas Model 2 also included the following covariates: age, gender, ethnicity, BMI, income adequacy, injury severity, hospitalisation, threat of disability and access to health care services. To examine the impact of the inclusion of the covariates, estimates obtained from Model 2 were compared to those from a modified Model 1 that was restricted to the same 1,753 participants (not presented). As the change in the coefficients was minimal, the following is based on the Model 1.

**Table 4:** GEE model output for rate of change of disability (WHODAS≥10) in the 24 months following injury (estimated from interviews conducted at 3, 12 and 24 months).

Variable	Model 1: Includes adjustment for pre-injury WHODAS only (N = 1,862)			Model 2: Includes adjustment for covariates^a^ (N = 1,753)		
	Coeff.	95% CI	p-value	Coeff.	95% CI	p-value
Time^b^	-0.2866	(-0.3202, -0.2531)	<0.0005	-0.2911	(-0.3269, -0.2553)	<0.0005
Quadratic term:						
(Time)^2^	0.0074	(0.0062, 0.0085)	<0.0005	0.0075	(0.0063, 0.0087)	<0.0005
Pre-existing comorbidity:						
None	ref			ref		
One comorbidity	0.0304	(-0.2123, 0.2732)	0.8	0.0565	(-0.1990, 0.3121)	0.7
Multimorbidity	0.3113	(0.0428, 0.5797)	0.02	0.2684	(-0.0196, 0.5563)	0.07
Interaction term - (Pre-existing comorbidity)*(Time):						
None	ref			ref		
One comorbidity	0.0043	(-0.0141, 0.0226)	0.6	-0.0003	(-0.0196, 0.0203)	1.0
Multimorbidity	0.0221	(0.0042, 0.0400)	0.02	0.0231	(0.0039, 0.0424)	0.02

^a^Covariates were age, gender, ethnicity, BMI, income adequacy, access to health care services, injury severity, hospitalised for injury, threat of disability and pre-injury WHODAS. 85 participants had item-missingness in at least one of these variables.

^b^Days between date of injury and date of interview

Including two variables to model the effect of time appear justified and provide evidence regarding difference in rates of recovery by pre-injury comorbidity (Table 4). Model 1 estimated no difference (log odds per year 0.052, 95%CI -0.169 to 0.271) between the rate of change of disability for those with one pre-injury comorbidity compared to those with none. In comparison, those with pre-injury multimorbidity had a significantly slower reduction in disability than those with no comorbidity (log odds per year 0.265, 95%CI 0.050 to 0.480). Comparison of the observed and predicted percentage of participants experiencing disability over time indicated acceptable model fit.

**Fig 1:**
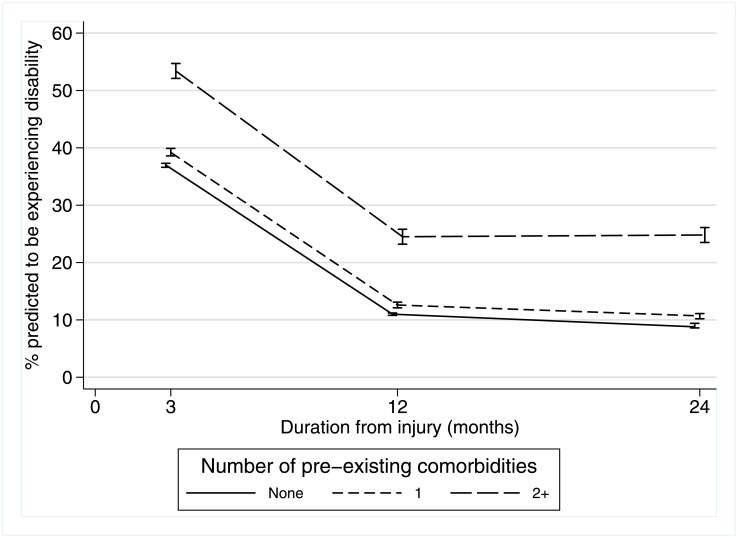
Estimated rates of recovery over time: Disability (WHODAS≥10) predictions for 24 months following injury by pre-existing comorbidity groups.

From 3 months to 12 months, of those with no comorbidity the percent predicted to be experiencing disability reduced from 37% to 11% (a 70% reduction) compared to a 54% reduction for the multimorbidity group (Fig 1). In the 12–24 month post-injury period, the percentage experiencing disability was relatively stable (increased from 24.5% to 24.8%) for the multimorbidity group whereas a reduction was observed for those with no comorbidity.

Whether the pre-injury comorbidities were physical or mental health conditions was examined as a possible explanation for differences in outcome for those with prior multimorbidity. Of those with one pre-injury comorbidity, 464 (91%) reported a physical comorbidity and 46 (9%) a mental comorbidity; of those with multimorbidity, 231 (59%) reported only physical pre-injury comorbidities, 21 (5%) reported only mental pre-injury comorbidities with the remaining 143 (36%) reporting both physical and mental pre-injury comorbidities.

The average recovery pattern over time for those with multiple physical comorbidities was similar to that presented in Fig 1 for those with multiple comorbidities (including both physical and mental). Rather than the parallel recovery patterns observed in Fig 1 for those with none and one comorbidity, those with only one physical comorbidity had the same prevalence of disability at 24 months as the group with no comorbidity despite starting at a higher prevalence at 3 months post injury.

## Discussion

The finding that for these entitlement claimants there was no evidence of a difference in recovery rates, as measured by lack of disability, in the 24 months following injury between those with no pre-injury comorbidities and those with one comorbidity is thought-provoking as is the finding that the rate of recovery was significantly slower for those with multimorbidity compared to those with no prior comorbid conditions. The first of these findings supports earlier research conducted using functional outcomes [10] and return to work/study to assess recovery [10, 11]. Unlike Polinder that reported considerable improvement between 9 months and 24 months for those with multimorbidity, POIS participants with multimorbidity reported minimal change in disability between 12 months and 24 months.

Almost all of the POIS cohort (2626 of 2856; 92%) were workforce active at the time of their injury indicating that pre-existing chronic conditions were able to be managed sufficiently so as not to obstruct functioning. This ability to function despite having a chronic condition could explain why those with one pre-existing comorbidity recover from injury at a similar rate to those with none. For those with pre-existing multimorbidity, the addition of an injury may be more likely to cause people to reach a ‘tipping point’, overwhelmed with difficulties in everyday management and engagement with rehabilitation thus leading to worse recovery rates. Adding weight to this ‘tipping point’ theory is previous research that suggests the relationship between increasing numbers of chronic diseases and disability is exponential rather than additive [27].

Different patterns in the rates of recovery were apparent in the 0–12 month and 12–24 month post-injury periods for those with pre-existing multimorbidity compared to others. One possible explanation for this could be that system and professional-related issues with healthcare delivery that have been reported for patients with multimorbidity deter those managing multimorbidity and an injury from sustaining longer-term engagement with rehabilitation services [28]. Future studies should explore this possibility further and include more frequent outcome measurements, particularly within the first year post-injury, to increase understanding of why, and when, recovery rates for those with multimorbidity worsen.

Analysis of a subset of participants that only reported physical comorbidities (no mental comorbidities) pre-injury found the same pattern of recovery rates by comorbidity group as for the entire cohort. Interestingly, the estimates suggest that the difference in recovery rates between those with two or more physical-only comorbidities and those with no pre-existing comorbidities may be larger than that reported for the full cohort although further research into the impact of physical and mental comorbidities is needed.

### Strengths and limitations

Varying definitions of comorbidity exist [29]. The prevalence of pre-injury comorbidity observed in this study is influenced by the ‘working age’ cohort recruited to POIS. Future studies should explore whether these findings extend to those 65 years and older who are likely to have higher rates of comorbidities.

Among those reporting multimorbidity at the time of injury, there will be heterogeneity. No information was collected on the severity of each comorbidity apart from that it had been diagnosed by a doctor and was likely to last for more than six months [18]. In addition no information was available on how well the comorbidity was being managed. A condition like asthma, for example, that is kept ‘under control’ through lifestyle and medication is less likely to impede recovery than a condition like Myalgic Encephalopathy/Chronic Fatigue Syndrome.

Loss to follow up is always a limitation of longitudinal studies. Conclusions here are based on participants that completed each of the 3, 12 and 24 month interviews. Previous POIS analyses examining factors associated with non-participation reported men, young adults, indigenous people and those living with people other than family members were more likely not to participate in follow-up interviews [30]. This, combined with the characteristics of those not included in this complete case analysis, suggests lost to follow could be associated with disability thus it may have some influence on our results.

Recovery was assessed using a measure of disability–the WHODAS that was developed specifically to link at the level of the concepts to the International Classification of Functioning, Disability and Health [15]. Use of a validated measure of disability is rare in injury outcome studies and is thus a strength of this study.

## Conclusions

In a ‘working age’ cohort, the rate of recovery in the 24 months following injury was similar for those with none or one pre-existing comorbidity and significantly slower for those with multimorbidity. Given the increasing prevalence of multimorbidity in many countries, [5] it is important that further research explores the mechanisms driving this finding and that health providers look at ways to better support those with multimorbidity following an injury.

## Supporting information

S1 Table POIS participants included in analysis compared to those lost to follow up or with item-missingness. (DOCX)
