# Supplementary material for: The role of pre-existing comorbidity on the rate of recovery following injury: A longitudinal cohort study
Source: PLoS One. 2018 Feb 21;13(2):e0193019. doi: 10.1371/journal.pone.0193019 (PMC5821361; doi:10.1371/journal.pone.0193019)
Supplement: S1 Table — (DOCX) [file pone.0193019.s001.docx]

| **S1 Table.** | | | | | | | |
| --- | --- | --- | --- | --- | --- | --- | --- |
|  |  |  |  |  |  |  |  |
|  |  |  |  |  |  |  |  |
|  | Included in complete case analysis (N=1,862) | |  | Not included in complete case analysis (N=994) | |  | p-value* |
|  | n | % |  | n | % |  |  |
|  |  |  |  |  |  |  |  |
| Age (years) |  |  |  |  |  |  |  |
| 18-24 | 200 | 10.7 |  | 196 | 19.7 |  | <0.01 |
| 25-34 | 354 | 19.0 |  | 239 | 24.0 |  |  |
| 35-44 | 417 | 22.4 |  | 213 | 21.4 |  |  |
| 45-54 | 495 | 26.6 |  | 208 | 20.9 |  |  |
| 55-64 | 396 | 21.3 |  | 138 | 13.9 |  |  |
| Sex |  |  |  |  |  |  |  |
| Male | 1081 | 58.1 |  | 672 | 67.6 |  | <0.01 |
| Female | 781 | 41.9 |  | 322 | 32.4 |  |  |
| BMI |  |  |  |  |  |  |  |
| <18.5 | 19 | 1.0 |  | 14 | 1.4 |  |  |
| 18.5 to <25 | 632 | 33.9 |  | 341 | 34.3 |  | <0.01 |
| 25 to <30 | 699 | 37.5 |  | 341 | 34.3 |  |  |
| 30+ | 454 | 24.4 |  | 230 | 23.1 |  |  |
| not disclosed | 58 | 3.1 |  | 68 | 6.8 |  |  |
| Ethnicity (prioritised) |  |  |  |  |  |  |  |
| NZ European | 1127 | 60.6 |  | 423 | 42.8 |  |  |
| Maori | 310 | 16.7 |  | 256 | 25.9 |  | <0.01 |
| Pacific | 88 | 4.7 |  | 115 | 11.6 |  |  |
| Other | 336 | 18.1 |  | 195 | 19.7 |  |  |
| Income adequacy |  |  |  |  |  |  |  |
| yes | 1688 | 90.7 |  | 865 | 87.0 |  |  |
| no | 162 | 8.7 |  | 108 | 10.9 |  | 0.04 |
| missing | 12 | 0.6 |  | 21 | 2.1 |  |  |
| Injury severity |  |  |  |  |  |  |  |
| low (NISS 1 to 3) | 751 | 40.3 |  | 442 | 44.5 |  |  |
| medium (NISS 4 to 6) | 860 | 46.2 |  | 428 | 43.1 |  | 0.08 |
| high (NISS >6) | 195 | 10.5 |  | 91 | 9.2 |  |  |
| missing | 56 | 3.0 |  | 33 | 3.3 |  |  |
| Preinjury disability |  |  |  |  |  |  |  |
| no (whodas<10) | 1769 | 95.0 |  | 907 | 91.2 |  |  |
| yes (whodas 10+) | 93 | 5.0 |  | 53 | 5.3 |  | 0.6 |
| missing | 0 | 0.0 |  | 34 | 3.4 |  |  |
| Threat of disability |  |  |  |  |  |  |  |
| yes | 713 | 38.3 |  | 454 | 45.7 |  |  |
| no | 1119 | 60.1 |  | 511 | 51.4 |  | <0.01 |
| missing | 30 | 1.6 |  | 29 | 2.9 |  |  |
| Access to health services |  |  |  |  |  |  |  |
| no problems | 1670 | 89.7 |  | 870 | 87.5 |  |  |
| problems | 176 | 9.5 |  | 112 | 11.3 |  | 0.1 |
| missing | 16 | 0.9 |  | 12 | 1.2 |  |  |
| Hospitalised |  |  |  |  |  |  |  |
| no | 471 | 33.9 |  | 240 | 31.8 |  |  |
| yes | 1391 | 100.0 |  | 754 | 100.0 |  | 0.5 |
| Preexisting comorbidities |  |  |  |  |  |  |  |
| 0 | 957 | 51.4 |  | 475 | 47.8 |  |  |
| 1 | 510 | 27.4 |  | 248 | 24.9 |  | 0.5 |
| 2+ | 395 | 21.2 |  | 173 | 17.4 |  |  |
| missing | 0 | 0.0 |  | 98 | 9.9 |  |  |
| * Those with missing information were excluded from the Chi-square test | | | | | |  |  |
